# Digital identification and adulteration analysis of Codonopsis Radix and Stellariae Radix based on the “digital identity” of chemical compositions

**DOI:** 10.3389/fchem.2024.1438321

**Published:** 2024-11-07

**Authors:** Xianrui Wang, Jiating Zhang, Wenguang Jing, Xiaohan Guo, Minghua Li, Xianlong Cheng, Feng Wei

**Affiliations:** Institute for Control of Traditional Chinese Medicine and Ethnic Medicine, National Institutes for Food and Drug Control, Beijing, China

**Keywords:** adulteration analysis, digital identification, Codonopsis Radix, Stellariae Radix, digital identity, chemical analysis

## Abstract

**Introduction:**

Under the background of digitalization of traditional Chinese medicine (TCM), this study aimed to realize the digital identification and adulteration analysis of Codonopsis Radix (CR) and Stellariae Radix (SR) based on chemical analysis.

**Methods:**

This study combined digitalization concepts and chemical analysis and conducted a chemical analysis of CR and SR from different batches based on UHPLC-QTOF-MS^E^. Furthermore, the shared ions were extracted from different batches of CR and SR as their “ion characterization” after digital quantization. Then, the data matrices of unique ions of CR relative to SR and SR relative to CR were screened out, and the top-N ions were outputted as the “digital identities” of CR and SR, sorted by ionic strength. Finally, the above “digital identities” of CR and SR were used as benchmarks for matching positive samples and market samples to provide feedback on the matching credibility (MC) for identification and adulteration analysis.

**Results:**

The results showed that based on the “digital identities” of CR and SR, the digital identification of CR, SR, and positive samples can be realized at the individual level of TCM efficiently and accurately, even if 3% of SR in the mixed samples can still be identified efficiently and accurately. Moreover, 1 of the 12 batches of market samples was identified as an adulterated sample.

**Conclusion:**

It proved that the identification and adulteration analysis of two herbs can be realized efficiently and quickly through the “digital identities” of chemical compositions. It has important reference significance for developing the digital identification of CR and SR at the individual level of Chinese medicine based on the “digital identity” of chemical compositions, which was beneficial to the construction of digital quality control of CR and SR.

## 1 Introduction

Codonopsis Radix (CR) is the dried root of *Codonopsis pilosula* (Franch.) Nannf., *Codonopsis pilosula* Nannf. var. *modesta* (Nannf.) L.T. Shen, or *Codonopsis tangshen* (Oliv.), family Eustoma (Liang et al., 2024). It is efficient in strengthening the spleen, benefiting the lungs, nourishing blood, and promoting the production of fluids, and is commonly used in the clinic for spleen and lung qi deficiency, low food intake, and lethargy (Liang et al., 2024; Dong et al., 2023). Stellariae Radix (SR) is the dried root of *Stellaria dichotoma* L.var. *lanceolata* Bge, family Caryophyllaceae (Wu et al., 2024). It is efficient in clearing deficiency heat and removing chancre heat and is commonly used clinically for yin deficiency fever, bone vapor, and labor heat (Wu et al., 2024; Li et al., 2017). Although the two herbs belong to different plant families and their effects and clinical pharmacological actions are different from each other, the medicinal parts of CR and SR are all dried roots and have similar traits and microscopic features such as cylindrical shape, yellowish brown surface with longitudinal wrinkles, more than 10 rows of cork cells, and sieve tube clusters (National Pharmacopoeia Commission, 2020). Therefore, it is very easy to confuse CR and SR in the herb market and clinical use. On the other hand, in recent years, because the market price of CR has been increasing continuously, in pursuit of higher interests, some unscrupulous businessmen pretend to sell SR as CR or mix SR into CR, which seriously impacts medication safety and medication effect. So, to strengthen the market supervision and quality control, it is important to realize the identification and adulteration analysis of CR and SR.

To realize the identification and adulteration analysis of CR and SR, relevant scholars have conducted a series of research. For example, Chen HJ used morphological and microscopic methods to identify CR and SR, but the methods need the support of professional technology (Chen, 2016). Wang S et al. identified CR by partial least square discriminant analysis based on the fusion data on electronic tongue and electronic nose (Wang et al., 2022). To find the genetic markers of CR, He JY et al. conducted the molecular analysis of the internal transcribed spacer sequence of nuclear ribosomal DNA of CR and so on (He et al., 2014). All of the above studies are beneficial to the identification analysis and quality control of CR. However, the identification analysis based on traits and microscopy requires a high degree of specialized knowledge and is highly subjective. In addition, the DNA, traits, and microscopic characteristics are highly susceptible to destruction during sample preparation (Hou et al., 2023). On the other hand, compared with DNA, traits, and microscopic characteristics, the chemical compositions are very transferable and tend not to be greatly damaged. So, it is very good to carry out the identification analysis of CR and SR from the perspective of chemical compositions. However, the current thin-layer chromatography and quality control markers focus on known chemical components and fail to utilize the information about unknown chemical components reasonably and effectively. In addition, the current identification analysis based on chemical composition focuses more on the discrimination between two herbs, rather than on the adulteration analysis.

Given the above considerations, as well as in the era of digitization and informatization of traditional Chinese medicine (TCM) (Xie et al., 2010; Wang et al., 2021), this study integrated digitization into chemical analysis and carried out the digital identification and adulteration analysis of CR and SR from the perspective of chemical analysis to strengthen the market regulation and quality control of CR and provide reference and assistance in the digital identification of TCM based on chemical constituents. First, UHPLC-QTOF-MS^E^ was used to determine CR and SR to obtain mass spectral information reflecting chemical constituents. Furthermore, the mass spectral information about CR and SR was converted to data matrices containing retention time (Rt), the mass-to-charge ratio (m/z), and ionic strength (I) using Progenesis QI software and msConvert. Then, the shared ions were extracted from different batches of CR and SR control medicinal materials as their “data characterization.” Then, the data matrices of unique ions of CR relative to SR and SR relative to CR were screened out, and the top-N ions were outputted as the “digital identities” of CR and SR, which were sorted by ionic strength. Finally, the above “digital identities” of CR and SR were used as a benchmark for matching positive samples and market samples to provide feedback on matching credibility (MC). The digital identification of CR and SR can be realized according to the MC of samples compared with “digital identities”.

## 2 Materials and methods

### 2.1 Herbal materials

Six batches of reference herbs of CR were obtained from the National Institutes for Food and Drug Control (NIFDC), and six batches reference herbs of SR were obtained from Gansu Institutes for Food and Drug Control (GSIFDC). Nine batches of homemade positive mixed samples, namely, 0% SR, 3% SR, 5% SR, 10% SR, 20% SR, 30% SR, 40% SR, 50% SR, and 100% SR were prepared by laboratory researchers. In addition, 12 batches of market CR samples were obtained from the Hebei Anguo herbal market. The detailed information about 33 batches of herbal materials such as the lot number, year, name, and use is given in Supplementary Table S1.

### 2.2 Reagent and laboratory consumables

The formic acid (MS grade, lot: L1670) and methanol (MS grade, lot: ED341-CN) were purchased from Honeywell Trading Co., Ltd of Shanghai China. Ultra-high-purity water was prepared using a Milli-Q water purification system (Millipore, Bedford, MA, United States). Leucine enkephalin (LE) was purchased from Sigma-Aldrich (St. Louis, MO, United States), and acetonitrile (MS grade, lot: 222372) was purchased from Thermo Fisher Scientific Technology Co., Ltd of Shanghai China. Syringe filters (0.22 μm) were purchased from Millipore (Billerica, MA, United States), and the 1.00-mL disposable syringe was purchased from Shandong Gaowei Technology Co., Ltd. The 2.00-mL injection vials were purchased from Waters Co., Ltd.

### 2.3 UHPLC-QTOF-MS^E^ analytical conditions

The UHPLC-QTOF-MS^E^ analysis was performed using ultra-high-performance liquid chromatography-tandem time-of-flight mass spectrometry on Waters Xevo G2-XS QTOF (Waters, United States) and Waters Acquity^TM^ UPLC systems (Waters MS Technologies, Manchester, United Kingdom). At the same time, the chromatographic separations were conducted on a Waters Acquity UPLC BEH-C_18_ (2.1 mm × 100 mm, 1.7 μm) chromatographic column (lot: 186002352, Waters, United States), while the column temperature was maintained at 35 °C. The mobile phase used for the elution consisted of 0.1% (v/v) formic acid in ultrapure water (solvent A) and acetonitrile (solvent B), and gradient elution was performed as follows: 0.00–23.00 min, 5%–95% B; 23.00–26.00 min, 95% B; 26.00–26.10 min, 95%–5% B; and 26.10–30.00 min, 5% B. The flow rate was set at 0.3 mL/min with an injection volume of 2 μL, and the auto-sampler was maintained at 25 °C. On the other hand, Waters mass spectrometry adopts the ESI positive ion mode and MS^E^ data acquisition method, in which the data acquisition rate was set to 0.2 s. The scanning range of *m/z* was 50–1,500. The collision gas was high-purity argon, and the real-time mass axis calibration solution (lock mass) was LE, of which the concentration was 500 ng/mL. In addition, capillary: 3.0 kV; sampling cone: 40 V; source offset: 80 V; desolvation temperature: 400 °C; desolvation gas: 800 L/h; collision energy: 0–40 V; and source temperature: 120 °C. Before sample analysis, the mass axis and lock mass were calibrated.

### 2.4 Sample preparation

For the preparation of samples, first, all of the herbs were powdered to filter using a No. 3 sieve. Then, the CR reference sample was prepared by mixing all batches of CR powder in equal proportions. In contrast, the SR reference sample was prepared by mixing all batches of SR powder in equal proportions. Furthermore, different proportions of SR reference sample powder were added to the CR reference sample powder (0%, 3%, 5%, 10%, 20%, 30%, 40%, 50%, and 100%) to prepare mixed positive samples. Finally, 1.0 g powder of CR, SR, mixed positive samples, and market herbs were accurately weighed and placed in a 50-mL tapered bottle with a plug; at the same time, 50.00 mL of methanol (MS grade) was accurately added using a pipette to a tapered bottle to perform ultrasound for 30 min (power: 500 W; frequency: 40 kHz). After taking out and cooling to room temperature, as well as filtering using a 0.22-μm organic filter membrane to obtain the analysis samples, all samples were stored at 4 °C in the refrigerator before the UHPLC-QTOF-MS^E^ analysis.

### 2.5 Data processing and identification algorithm flow

The mass spectrogram of CR, SR, mixed positive samples, market CRs, and blank solvent (methanol) was processed using Progenesis QI and msConvert to convert into data matrices (TCM-DM) of [*Rt-m/z-I*] (Sun et al., 2021). At the same time, the peak picking limits were automatic, and the retention time was limited to 1.00–26.00 min. So we can obtain the quantized data on each sample including the retention time (*Rt*), the mass-to-charge ratio (*m/z*), and ionic strength (*I*) of primary ions and secondary fragment ions. Furthermore, the quantized data on primary ions and secondary fragment ions were uniformly integrated and saved as a CSV file in the following form:

$$\text{Blank or TCM}-\text{DIC }\left(\text{name}\right)=\left[\begin{array}{ccc} Rt & m/z & I\\ ••• & ••• & •••\\ t & m & i \end{array}\right].$$

On the other hand, the specific identification algorithm flow can be decomposed into the following steps:

(1) Interfering ion elimination: To remove interfering ions originating from blank (methanol) in all samples, the procession would judge whether the ions in samples come from blank methanol and delete the background ions using the following criteria: the ions in the samples and methanol have similar *Rt* and *m/z* (Δ*Rt* ≤ 0.10 min and Δ*m/z* ≤ 0.01 Da). The specific algorithm for this section is as follows.

Assuming that the digital matrices of three samples (CR_1_, CR_2_, and CR_3_) of CR in different batches are recorded as *C*_1_, *C*_2_, and *C*_3_ respectively, the digital matrices of three samples (SR_1_, SR_2_, and SR_3_) of SR in different batches are recorded as *S*_1_, *S*_2_, and *S*_3_, respectively, and the digital matrices of the corresponding blank methanol (BM_1_, BM_2_, and BM_3_) are recorded as *B*_1_, *B*_2_, and *B*_3_, respectively,

$$C_{1}=\left[\begin{array}{ccc} Rt & m/z & I\\ ••• & ••• & •••\\ t_{C1} & m_{C1} & i_{C1} \end{array}\right]\;C_{2}=\left[\begin{array}{ccc} Rt & m/z & I\\ ••• & ••• & •••\\ t_{C2} & m_{C2} & i_{C2} \end{array}\right]\;C_{3}=\left[\begin{array}{ccc} Rt & m/z & I\\ ••• & ••• & •••\\ t_{C3} & m_{C3} & i_{C3} \end{array}\right],$$

$$S_{1}=\left[\begin{array}{ccc} Rt & m/z & I\\ ••• & ••• & •••\\ t_{S1} & m_{S1} & i_{S1} \end{array}\right]\;S_{2}=\left[\begin{array}{ccc} Rt & m/z & I\\ ••• & ••• & •••\\ t_{S2} & m_{S2} & i_{S2} \end{array}\right]\;S_{3}=\left[\begin{array}{ccc} Rt & m/z & I\\ ••• & ••• & •••\\ t_{S3} & m_{S3} & i_{S3} \end{array}\right],$$

$$B_{1}=\left[\begin{array}{ccc} Rt & m/z & I\\ ••• & ••• & •••\\ t_{B1} & m_{B1} & i_{B1} \end{array}\right]\;B_{2}=\left[\begin{array}{ccc} Rt & m/z & I\\ ••• & ••• & •••\\ t_{B2} & m_{B2} & i_{B2} \end{array}\right]\;B_{3}=\left[\begin{array}{ccc} Rt & m/z & I\\ ••• & ••• & •••\\ t_{B3} & m_{B3} & i_{B3} \end{array}\right].$$

Furthermore, the new data matrices *M*_1_, *M*_2_, and *M*_3_ obtained based on the interfering ion data in the corresponding blank matrices are eliminated from the data matrices *C*_1_, *C*_2_, and *C*_3_ of CR:

$$M_{1}=C_{1}-C_{1}\cap B_{1}=\left[\begin{array}{ccc} Rt & m/z & I\\ ••• & ••• & •••\\ t_{C1} & m_{C1} & i_{C1} \end{array}\right]-\left[\begin{array}{ccc} Rt & m/z & I\\ ••• & ••• & •••\\ t_{C1} & m_{C1} & i_{C1} \end{array}\right]\cap \left[\begin{array}{ccc} Rt & m/z & I\\ ••• & ••• & •••\\ t_{B1} & m_{B1} & i_{B1} \end{array}\right]=\left[\begin{array}{ccc} Rt & m/z & I\\ ••• & ••• & •••\\ t_{M1} & m_{M1} & i_{M1} \end{array}\right],$$

$$M_{2}=C_{2}-C_{2}\cap B_{2}=\left[\begin{array}{ccc} Rt & m/z & I\\ ••• & ••• & •••\\ t_{C2} & m_{C2} & i_{C2} \end{array}\right]-\left[\begin{array}{ccc} Rt & m/z & I\\ ••• & ••• & •••\\ t_{C2} & m_{C2} & i_{C2} \end{array}\right]\cap \left[\begin{array}{ccc} Rt & m/z & I\\ ••• & ••• & •••\\ t_{B2} & m_{B2} & i_{B2} \end{array}\right]=\left[\begin{array}{ccc} Rt & m/z & I\\ ••• & ••• & •••\\ t_{M2} & m_{M2} & i_{M2} \end{array}\right],$$

$$M_{3}=C_{3}-C_{3}\cap B_{3}=\left[\begin{array}{ccc} Rt & m/z & I\\ ••• & ••• & •••\\ t_{C3} & m_{C3} & i_{C3} \end{array}\right]-\left[\begin{array}{ccc} Rt & m/z & I\\ ••• & ••• & •••\\ t_{C3} & m_{C3} & i_{C3} \end{array}\right]\cap \left[\begin{array}{ccc} Rt & m/z & I\\ ••• & ••• & •••\\ t_{B3} & m_{B3} & i_{B3} \end{array}\right]=\left[\begin{array}{ccc} Rt & m/z & I\\ ••• & ••• & •••\\ t_{M3} & m_{M3} & i_{M3} \end{array}\right].$$

On the other hand, for the data matrices of SRs, we can obtain the new data matrices *N*_1_, *N*_2_, and *N*_3_ excluding the blank ion interference:

$$N_{1}=S_{1}-S_{1}\cap B_{1}=\left[\begin{array}{ccc} Rt & m/z & I\\ ••• & ••• & •••\\ t_{S1} & m_{S1} & i_{S1} \end{array}\right]-\left[\begin{array}{ccc} Rt & m/z & I\\ ••• & ••• & •••\\ t_{S1} & m_{S1} & i_{S1} \end{array}\right]\cap \left[\begin{array}{ccc} Rt & m/z & I\\ ••• & ••• & •••\\ t_{B1} & m_{B1} & i_{B1} \end{array}\right]=\left[\begin{array}{ccc} Rt & m/z & I\\ ••• & ••• & •••\\ t_{N1} & m_{N1} & i_{N1} \end{array}\right],$$

$$N_{2}=S_{2}-S_{2}\cap B_{2}=\left[\begin{array}{ccc} Rt & m/z & I\\ ••• & ••• & •••\\ t_{S2} & m_{S2} & i_{S2} \end{array}\right]-\left[\begin{array}{ccc} Rt & m/z & I\\ ••• & ••• & •••\\ t_{S2} & m_{S2} & i_{S2} \end{array}\right]\cap \left[\begin{array}{ccc} Rt & m/z & I\\ ••• & ••• & •••\\ t_{B2} & m_{B2} & i_{B2} \end{array}\right]=\left[\begin{array}{ccc} Rt & m/z & I\\ ••• & ••• & •••\\ t_{N2} & m_{N2} & i_{N2} \end{array}\right],$$

$$N_{3}=S_{3}-S_{3}\cap B_{3}=\left[\begin{array}{ccc} Rt & m/z & I\\ ••• & ••• & •••\\ t_{S3} & m_{S3} & i_{S3} \end{array}\right]-\left[\begin{array}{ccc} Rt & m/z & I\\ ••• & ••• & •••\\ t_{S3} & m_{S3} & i_{S3} \end{array}\right]\cap \left[\begin{array}{ccc} Rt & m/z & I\\ ••• & ••• & •••\\ t_{B3} & m_{B3} & i_{B3} \end{array}\right]=\left[\begin{array}{ccc} Rt & m/z & I\\ ••• & ••• & •••\\ t_{N3} & m_{N3} & i_{N3} \end{array}\right].$$

(2) Shared ion acquisition: The mass spectrometry data from different batches of CR and SR were used to extract their respective shared data and construct new data matrices, in which the shared ions in different batches of CR or SR samples had similar *Rt* and *m/z* (Δ*Rt* ≤ 0.10 min and Δ*m/z* ≤ 0.01 Da). The specific algorithm for this section is as follows.

The digital matrices of the shared ions of CR and SR are defined as *M* and *N*, respectively, which can be expressed as

$$M=M_{1}\cap M_{2}\cap M_{3}=\left[\begin{array}{ccc} Rt & m/z & I\\ ••• & ••• & •••\\ t_{M1} & m_{M1} & i_{M1} \end{array}\right]\cap \left[\begin{array}{ccc} Rt & m/z & I\\ ••• & ••• & •••\\ t_{M2} & m_{M2} & i_{M2} \end{array}\right]\cap \left[\begin{array}{ccc} Rt & m/z & I\\ ••• & ••• & •••\\ t_{M3} & m_{M3} & i_{M3} \end{array}\right]=\left[\begin{array}{ccc} Rt & m/z & I\\ ••• & ••• & •••\\ t_{M} & m_{M} & i_{M} \end{array}\right],$$

$$N=N_{1}\cap N_{2}\cap N_{3}=\left[\begin{array}{ccc} Rt & m/z & I\\ ••• & ••• & •••\\ t_{N1} & m_{N1} & i_{N1} \end{array}\right]\cap \left[\begin{array}{ccc} Rt & m/z & I\\ ••• & ••• & •••\\ t_{N2} & m_{N2} & i_{N2} \end{array}\right]\cap \left[\begin{array}{ccc} Rt & m/z & I\\ ••• & ••• & •••\\ t_{N3} & m_{N3} & i_{N3} \end{array}\right]=\left[\begin{array}{ccc} Rt & m/z & I\\ ••• & ••• & •••\\ t_{N} & m_{N} & i_{N} \end{array}\right].$$

(3) Proprietary ion acquisition: The data matrices of the specific ions of CR relative to SR were screened by comparing the CR’s shared data with the raw mass spectral data on SR samples. On the other hand, the data matrices of the specific ions of SR relative to CR were screened by comparing the SR’s shared data with the raw mass spectral data on CR samples. During the screening of the above characteristic ions, when the ions had similar *Rt* and *m/z* (ΔRt ≤ 0.20 min and Δm/z ≤ 0.30 Da), they would be taken as non-specific ions and be deleted. The specific algorithm for obtaining proprietary ions is explained herein.

We defined the digital matrices of proprietary ions of CR and SR as *X* and *Y*, respectively; at the same time, the union of *M*_1_, *M*_2_, and *M*_3_ was defined as *m* = (*M*_1_∪*M*_2_∪*M*_3_), and the union of *N*_1_, *N*_2_, and *N*_3_ was defined as *n* = (*N*_1_∪*N*_2_∪*N*_3_), which can be expressed as

$$m=M_{1}\cup M_{2}\cup M_{3}=\left[\begin{array}{ccc} Rt & m/z & I\\ ••• & ••• & •••\\ t_{M1} & m_{M1} & i_{M1} \end{array}\right]\cup \left[\begin{array}{ccc} Rt & m/z & I\\ ••• & ••• & •••\\ t_{M2} & m_{M2} & i_{M2} \end{array}\right]\cup \left[\begin{array}{ccc} Rt & m/z & I\\ ••• & ••• & •••\\ t_{M3} & m_{M3} & i_{M3} \end{array}\right]=\left[\begin{array}{ccc} Rt & m/z & I\\ ••• & ••• & •••\\ t_{m} & m_{m} & i_{m} \end{array}\right],$$

$$n=N_{1}\cup N_{2}\cup N_{3}=\left[\begin{array}{ccc} Rt & m/z & I\\ ••• & ••• & •••\\ t_{N1} & m_{N1} & i_{N1} \end{array}\right]\cup \left[\begin{array}{ccc} Rt & m/z & I\\ ••• & ••• & •••\\ t_{N2} & m_{N2} & i_{N2} \end{array}\right]\cup \left[\begin{array}{ccc} Rt & m/z & I\\ ••• & ••• & •••\\ t_{N3} & m_{N3} & i_{N3} \end{array}\right]=\left[\begin{array}{ccc} Rt & m/z & I\\ ••• & ••• & •••\\ t_{n} & m_{n} & i_{n} \end{array}\right],$$

$$X=M-n=M-\left(N_{1}\cup N_{2}\cup N_{3}\right)=\left[\begin{array}{ccc} Rt & m/z & I\\ ••• & ••• & •••\\ t_{M} & m_{M} & i_{M} \end{array}\right]-\left[\begin{array}{ccc} Rt & m/z & I\\ ••• & ••• & •••\\ t_{n} & m_{n} & i_{n} \end{array}\right]=\left[\begin{array}{ccc} Rt & m/z & I\\ ••• & ••• & •••\\ t_{X} & m_{X} & i_{X} \end{array}\right],$$

$$Y=N-m=Y-\left(M_{1}\cup M_{2}\cup M_{3}\right)=\left[\begin{array}{ccc} Rt & m/z & I\\ ••• & ••• & •••\\ t_{N} & m_{N} & i_{N} \end{array}\right]-\left[\begin{array}{ccc} Rt & m/z & I\\ ••• & ••• & •••\\ t_{m} & m_{m} & i_{m} \end{array}\right]=\left[\begin{array}{ccc} Rt & m/z & I\\ ••• & ••• & •••\\ t_{Y} & m_{Y} & i_{Y} \end{array}\right].$$

(4) “Digital identity” acquisition of CR and SR: Based on step (3), in the data matrices of X and Y, according to the ordering of ionic strengths from largest to smallest, the top 100 ions were retained as the “digital identity” for CR and SR, respectively.
(5) Digital match identification based on chemical compositions: The “digital identities” of CR and SR were taken as the benchmark for matching quantized data of CR, SR, positive mixed samples, and market herbs to obtain feedback on the MC. In the process of digital match identification, the criteria for digital matching were as follows: the ions in “digital identity” and samples to be identified had similar *Rt* and *m/z* (Δ*Rt* ≤ 0.10 min and Δ*m/z* ≤ 0.01 Da), and the formula of MC is as follows:

$$\text{MC}=\frac{\begin{array}{cccc} number & of & matched & ions \end{array}}{\begin{array}{cccc} number & of & ions & \begin{array}{ccc} in & digital & identity \end{array} \end{array}}\times 100\%.$$

The above algorithm flows were all written and implemented based on Java language.

## 3 Results

### 3.1 UHPLC-QTOF-MS^E^ analysis

**FIGURE 1:**
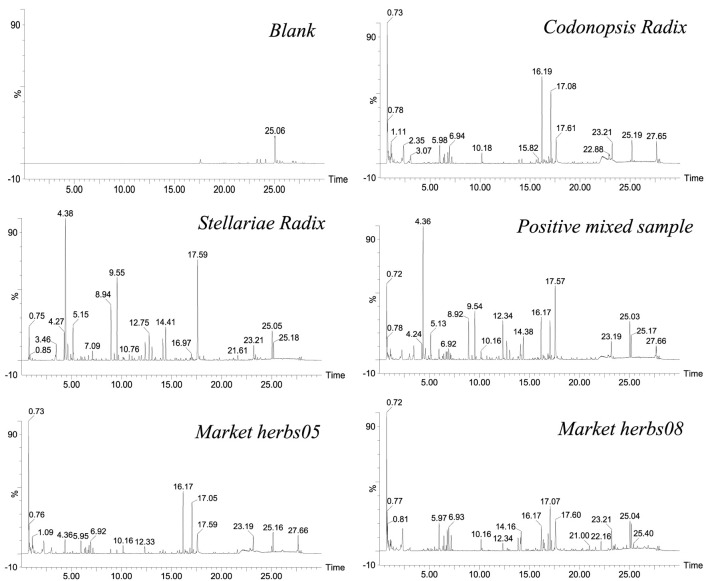
Base peak chromatograms of blank, CR, SR, positive mixed sample, and market herbs.

Through detection and analysis under the unified experimental conditions, we obtained the base peak chromatograms of CR, SR, positive samples, and market herbs. As shown in Figure 1, due to the difference in chemical compositions, CR and SR showed different base peak chromatograms. At the same time, it can be seen that blank methanol had no obvious interference with the detection of samples, and the base peak chromatograms of the positive mix sample and market herbs were relatively complicated. If only a single CR or SR is identified, it can be realized by base peak chromatogram comparison. However, with the increase in sample size and the prominence of individual differences, the efficiency will inevitably be low only by comparing the differences of base peak chromatograms. More importantly, it is difficult to effectively identify mixed positive samples and unknown market samples only by the base peak chromatograms of chemical components. Therefore, in the digital era of TCM, combining chemical composition analysis with digital concepts and carrying out digital identification analysis based on chemical compositions will help improve the analysis efficiency and realize the digital analysis of adulterated products.

### 3.2 Digital quantized processing and digital identity

In the process of detecting CR and SR, six blank methanol samples were collected at different periods. The six methanol samples were used as “blanks” to deduct background ions. After data conversion using Progenesis QI and msConvert, through harmonized integration and aggregation, they contained 4,099–4,301 [*Rt*-*m/z*-*I*] units with average value = 4,226 and RSD = 1.62%.

For CR and SR samples that were used to extract “digital identity,” the number of [*Rt*-*m/z*-*I*] units of CR and SR is shown in Table 1. Three different batches of CR and SR samples were detected at different times and selected to extract “digital identity,” and the different batches of CR or SR samples had different numbers of [*Rt*-*m/z*-*I*], which indicated the presence of different chemical compositions or different specific chemical fragments in the same herbal medicine of different origins, different years, and different batches. Conversely, even if the origin, years, and batch number are different, as long as it is the same Chinese medicine, there are bound to be the same chemical ingredients. Based on the above considerations, this study extracted and screened three batches of CR and SR samples for obtaining their respective “digital identities.” Finally, the top 100 [*Rt*-*m/z*-*I*] data matrices were screened as “digital identities” of CR and SR based on the ionic strength from largest to smallest. The results of the top 100 [*Rt*-*m/z*-*I*] data in “digital identities” of CR and SR are given in Supplementary Tables S2, S3.

**TABLE 1:** Number of [*Rt*-*m/z*-*I*] in CR and SR samples used to extract “digital identity.”

Herb	Batch	Number	Source	Herb	Batch	Number	Source
CR	CR01	9,672	NIFDC		SR02	7,488	GSIFDC
	CR03	9,487	NIFDC	SR	SR05	7,052	GSIFDC
	CR04	6,503	NIFDC		SR06	7,136	GSIFDC

### 3.3 Digital identification of CR and SR

Based on the “digital identities” of the chemical components of CR and SR, the digital identification of CR and SR was carried out. First, the test samples of CR and SR were analyzed by UHPLC-QTOF-MS^E^, and the mass spectrogram that reflected chemical composition information was transformed into the data matrices. Furthermore, the “digital identities” of CR and SR were taken as the benchmark to match the data matrices of test samples sequentially to obtain feedback on the MC. Finally, the identification of two herbs was realized according to MC. The results of the three test samples of CR are shown in Table 2.

**TABLE 2:** Matching credibility results of CR.

Herb	Batch	Match ions	Ions in the digital identity of CR or SR	MC (%)
CR	CR02	86	100—CR	86.00
	CR05	85	100—CR	85.00
	CR06	86	100—CR	86.00
	CR02	1	100—SR	1.00
	CR05	1	100—SR	1.00
	CR06	1	100—SR	1.00

As shown in Table 2, the MCs of CR samples compared with CR’s “digital identity” are all greater than 80.00%, in which the sample with batch number CR05 had the lowest MC of 85.00%. At the same time, the MCs of CR samples with batch numbers CR02, CR05, and CR06 compared with SR’s “digital identity” are all 1.00%. In other words, the MC between CR and its own “digital identity” is at least 80+ percentage points higher than the MC between CR and SR’s “digital identity.” On the other hand, the results of the three test samples of SR are shown in Table 3.

**TABLE 3:** Matching credibility results of SR.

Herb	Batch	Match ions	Ions in the digital identity of CR or SR	MC (%)
SR	SR01	100	100—SR	100.00
	SR03	99	100—SR	99.00
	SR04	103	100—SR	103.00
	SR01	0	100—CR	0.00
	SR03	0	100—CR	0.00
	SR04	0	100—CR	0.00

As shown in Table 3, the MCs of SR samples compared with SR’s “digital identity” are all greater than 95.00%, in which the sample with batch number SR03 had the lowest MC of 99.00%. At the same time, the MCs of SR samples with batch numbers SR01, SR03, and SR04 compared with CR’s “digital identity” are all 0.00%. In other words, the MC between CR and its own “digital identity” is at least 99+% points higher than the MC between CR and SR’s “digital identity.” In addition, the MC of the SR04 sample is 103.00%, which is greater than 100.00%. The above situation happened because of the existence of a matching deviation threshold, which led to the successful matching of multiple ions in the test sample with one ion in the SR’s “digital identity.” Furthermore, since the data were not normally distributed, the nonparametric rank-sum test was used to test whether there were significant differences between MCs. The results showed that the MCs of CR compared to the CR “digital identity” were significantly different from the MCs of CR compared to the SR “digital identity” ( p < 0.001 ). In addition, the 95% confidence interval for MCs of CR compared to CR’s “digital identity” was 85.0%–86.3% in the identification of single-herb CR. At the same time, the results also showed that the MCs of SR compared to the SR “digital identity” were significantly different from the MCs of SR compared to CR “digital identity” ( p < 0.0001 ). The 95% confidence interval for MCs of SR compared to SR’s “digital identity” was 98.3%–103.0% in the identification of single-herb SR.

After the nonparametric rank-sum test analysis, combined with Table 2, 3, the “digital identities” of chemical components of CR and SR have certain specificity. The digital identification of CR and SR can be effectively realized through the “digital identities” of CR and SR’s chemical components, as well as MC. There is a huge difference between the MC of samples compared with their own “digital identity” of chemical compositions and the MC of samples compared with the non-self “digital identity” of chemical compositions.

### 3.4 Adulterant identification analysis

#### 3.4.1 Identification of mixed positive samples

**FIGURE 2:**
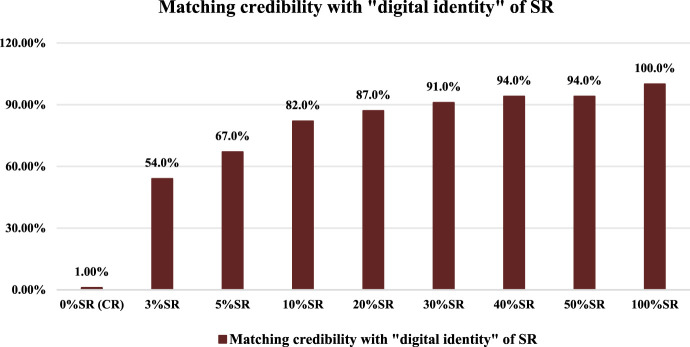
Matching results of mixed positive samples compared with SR’s “digital identity.”

In the same way, we take the “digital identities” of CR and SR’s chemical compositions as the benchmark to match nine batches of mixed positive samples (0% SR, 3% SR, 5% SR, 10% SR, 20% SR, 30% SR, 40% SR, 50% SR, and 100% SR). The matching results of mixed positive samples compared with SR’s “digital identity” are shown in Figure 2.

**FIGURE 3:**
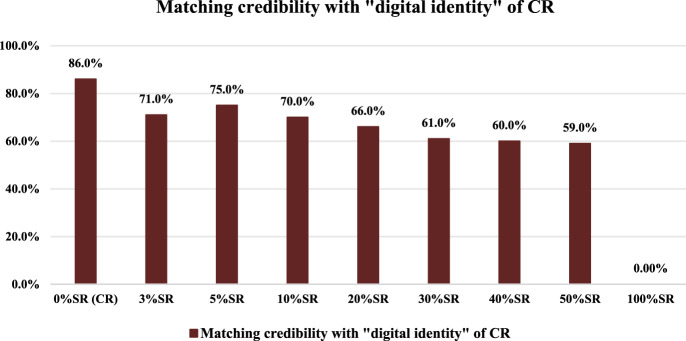
Matching results of mixed positive samples compared with CR’s “digital identity.”

As shown in Figure 2, with the increase in the SR’s proportion in mixed positive samples, the MCs of mixed positive samples compared with SR’s “digital identity” showed an obvious upward trend. From 3% SR and 5% SR to 50% SR and 100% SR, the MCs were all not less than 50.0%, in which the 3% SR had the smallest MC (54.0%). At the same time, the MC of 0% SR (CR reference sample) compared with SR’s “digital identity” was only 1.00%. In other words, the MC of 3% SR was 53% points higher than that of 0% SR (CR reference sample), which illustrated that the SR in mixed positive samples can be effectively identified based on the “digital identity” of SR’s chemical compositions and the matching credibility. In addition, the matching results of mixed samples compared with CR’s “digital identity” are shown in Figure 3.

As shown in Figure 3, on the whole, the MCs of mixed positive samples compared with CR’s “digital identity” showed a slight downward trend with the increase in the SR’s proportion. From 3% SR to 50% SR, the MCs of mixed positive samples compared with CR’s “digital identity” were all not less than 50.0%, with the 50% SR having the smallest MC value of 59.0%. On the other hand, it can be found that the MC of 50% SR was 59.0% and the MC of 100% SR was 0.00%, which reflected a huge difference in (MC. So the CR in mixed positive samples can be effectively identified based on the “digital identity” of CR’s chemical compositions and the matching credibility. In other words, adding SR to the CR sample did not affect the matching identification result between the CR sample and the “digital identity” of CR’s chemical compositions.

Further combined with Figures 2, 3, considering that 3% “impurities” are allowed in traditional Chinese medicine, we set the detection limit for matching credibility based on SR’s “digital identity” to 54.0%. In other words, when compared with the SR’s “digital identity,” if the MC of feedback was greater than 54.0%, the SR is considered to be detected in the corresponding samples.

#### 3.4.2 Digital identification of market CR samples

**FIGURE 4:**
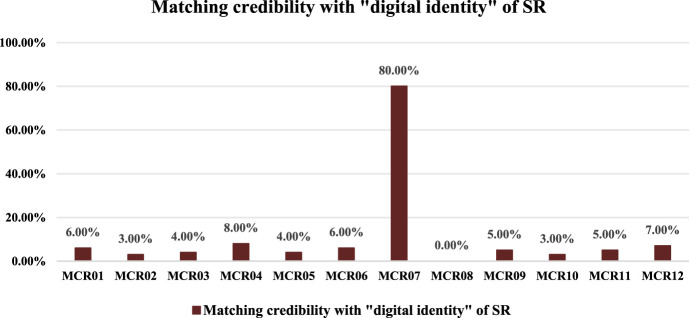
Matching results of market CR samples compared with SR’s “digital identity.”

In addition, based on the “digital identity” of CR and SR’s chemical compositions, we carried out the digital identification of CR medicinal materials on the Hebei Anguo herb market. The quantized information on the chemical compositions of market CR (MCR) samples was obtained based on the same sample processing, mass spectrometry detection, and digitization methods. Furthermore, the quantized data on market CR samples were compared with the “digital identity” of CR and SR’s chemical compositions. The results of the matching comparison are shown in Figure 4.

As shown in Figure 4, except for the MCR07 batch, the other market CR samples had MCs of less than 10.00% compared with the “identity identifiers” of SR’s chemical compositions, which were much less than the MC of SR’s detection limit (54.00%). This suggested that these batches of CR samples can be considered unadulterated with SR. On the other hand, for the MCR07 sample, the MC compared with the “digital identity” of SR’s chemical compositions was as high as 80.00% and was greater than 54.00% (detection limit). It showed that the market CR sample with batches of MCR07 was indeed adulterated with SR. At the same time, the MCs of market CR samples compared with “identity identifiers” of CR’s chemical compositions were in the range 47.00%–78.00%. Even if the MC was only 47.00%, MC is much higher than the MC of SR compared with the “digital identity” of CR’s chemical compositions. In summary, based on the “digital identities” of CR and SR’s chemical compositions, we identified 12 batches of market CR samples, of which the sample of MCR07 was a pseudo-product adulterated with SR, and the rest of them were genuine products with no SR detected.

#### 3.4.3 Chemical validation of digital identification

**FIGURE 5:**
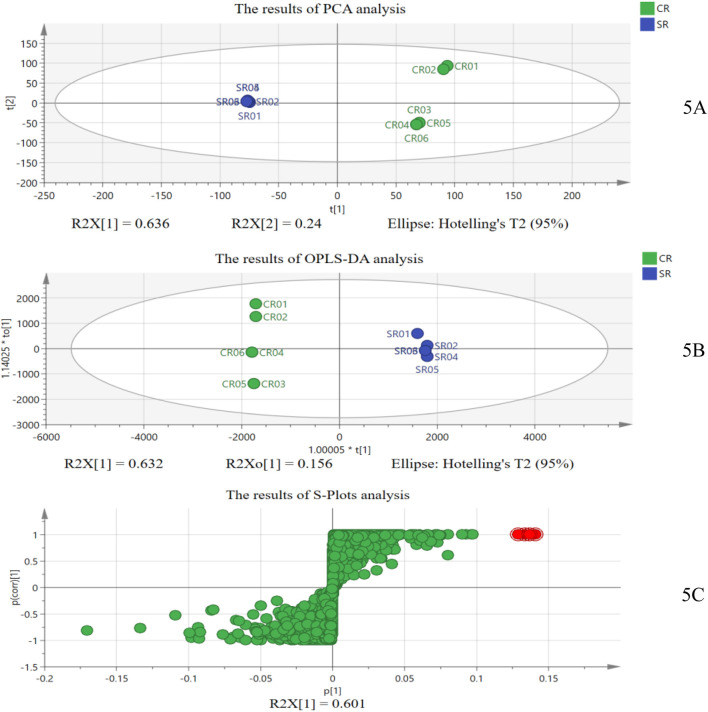
Results of chemometric analysis: **(A)** results of PCA; **(B)** results of OPLS-DA; and **(C)** results of S-plot analysis.

Furthermore, to prove the results of digital identification based on the “digital identity” of CR and SR’s chemical components, we also analyzed them based on traditional chemical analysis combined with chemometrics (Rebiai et al., 2022). First, taking the mixed sample of 50% SR as the quality control sample, the mass spectra of six batches of CRs and six batches of SRs were transformed into data pairs of exact mass retention time (EMRT), and the characteristic ions of SR were obtained through PCA, OPLS-DA, and S-plot analysis, which were used to identify whether CR samples were adulterated with SR (Ringnér, 2008; Kang et al., 2022; Cheng et al., 2017). The results of the chemometric analysis are shown in Figure 5.

**FIGURE 6:**
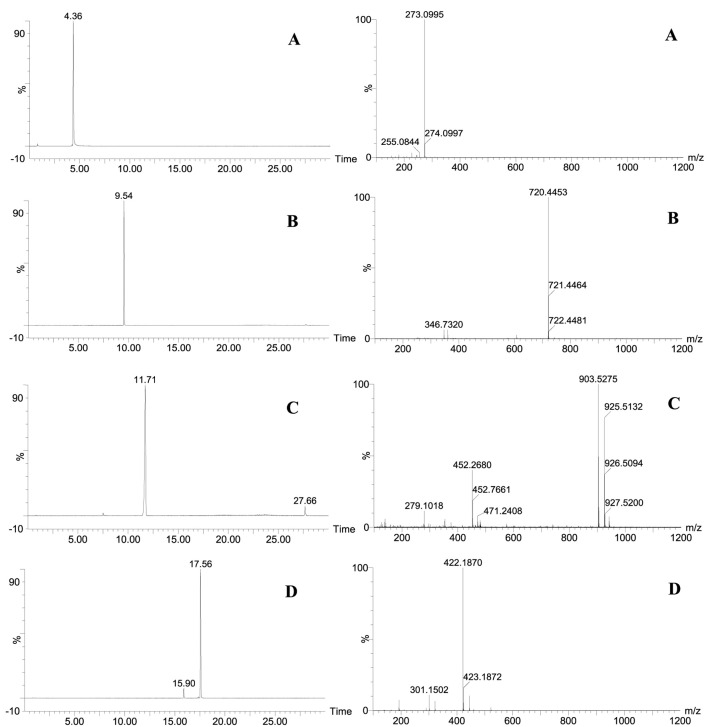
**(A)** Mass spectrometry information of chemical component A; **(B)** Mass spectrometry information of chemical component B; **(C)** Mass spectrometry information of chemical component C; **(D)** Mass spectrometry information of chemical component D.

As shown in Figure 5A, the SR samples can be effectively distinguished from CR samples at the overall level through unsupervised PCA with R^2^X = 0.909 and Q^2^ = 0.767, which showed that there are indeed different chemical components in SR and CR. Furthermore, the supervised OPLS-DA and S-plot analysis were used to explore the different chemical components between CR and SR. Figure 5B shows that the SR samples can also be effectively distinguished from CR samples with Q^2^ = 0.99 and the R^2^ = (0.0, 0.352), Q^2^ = (0.0, −0.797), and p=2.77 ×10^–8^ (<0.001) in model validation, which showed that the OPLS-DA model is accurate and reliable, with no over-fitting phenomenon. In the S-plot graph, the *x*-axis denotes the variable. The farther a data point that represents a chemical component is from the zero on the *x*-axis, the greater the contribution that the chemical component makes to the sample difference, which means that the EMRT data pairs (chemical components) of a high VIP value distributed at the two ends of the S-plots were regarded as the potential chemical markers. As shown in Figure 5C, the data points in the upper-right corner of the “S-curve” represent the chemical components in SR, especially the four red data points that are the proprietary chemical components (ions data) in SR and contribute the most to the difference between the CR and SR groups. As shown in Figure 6, these red data points are chemical component A (*Rt* 4.36 min; *m/z* 273.0995), chemical component B (*Rt* 9.54 min; *m/z* 720.4453), chemical component C (*Rt* 11.71 min; *m/z* 903.5275), and chemical component D (*Rt* 17.56 min; *m/z* 422.1870), which do not exist in CR samples.

Based on the above analysis, the chemical components A, B, C, and D were taken as proprietary chemical markers of SR that distinguish SR from CR, and the four chemical components were extracted from the mass spectrum information on CR and SR samples, mixed positive samples, and market CR samples. The results showed that the chemical components A, B, C, and D cannot be extracted from the CR samples; 0% SR, 11 batches of market CR samples (except MCR07), and their ionic strengths were basically at the blank baseline level. This means that there is no SR in CR samples, 0% SR, and 11 batches of market CR samples (except MCR07), which is consistent with the actual situation. In addition, the chemical components A, B, C, and D can be extracted from the SR samples, mixed positive samples (except 0% SR), and MCR07 sample, which indicates that SR was added to the MCR07 sample and is consistent with the results of digital identification based on the “digital identity” of CR and SR’s chemical components. Therefore, it can be concluded that the MCR07 sample is indeed adulterated with SR, and the digital identification based on the “digital identity” of the CR and SR chemical compositions is accurate and reliable through chemical verification.

## 4 Discussion

### 4.1 Optimization of analytical conditions

Before the chemical analysis of all samples, we explored and optimized the sample processing and detection conditions. During sample processing, we examined the extraction of pure water, methanol, and 50% methanol–water, and the results showed that more data information about chemical compositions was obtained when methanol was used as the extraction solvent. Furthermore, we examined the data when the sample powder was extracted by ultrasonication for 15 min, 30 min, and 45 min, and the results showed that the data information for 30 min and 45 min was basically the same, which was significantly higher than that for 15 min. Therefore, to ensure the amount of data and to save time, we chose to ultrasonicate for 30 min. On the other hand, in MS analysis, we first examined the positive and negative ion detection modes. The results showed that the ionic strength in the negative ion mode is much lower than that of the positive ion mode, resulting in the loss of a large amount of chemical composition data information during the data conversion process. Therefore, we finally chose the positive ion mode. Meanwhile, we adopted the MS^E^ data-independent acquisition mode in the experimental analysis. In this mode, the collision energy will change in gradient to obtain both the parent and secondary fragment ions, thus ensuring more data information. On the other hand, different traditional Chinese medicines will also produce unique characteristic fragment ions. Therefore, in this study, the secondary fragment ions and the primary parent ions were integrated into a CSV file with [*Rt*-*m/z*-*I*] format and further screened according to the ion intensity, so the top N ions were output as their “digital identity.” In addition, we investigated the mass spectrum information when the collision energies were 10 V–30 V, 10 V–40 V, and 10 V–50 V. It was distinct that the mass spectrum had the most abundant data information with the collision energy being 10 V–40 V, rather than 10 V–30 V or 10 V–50 V. This universal condition ensures the accuracy and repeatability of data collection in different periods.

In addition, for mass spectrometry data acquisition, more attention should be paid to the accuracy and reproducibility of the acquisition, rather than LOD, LOQ, and stability in quantitative analysis. The LE used in the experiment can correct the mass axis in real time to ensure the accuracy of the data. On the other hand, the mass spectrometry data on CR and SR were collected at different times, which can be found to have good reproducibility.

### 4.2 Optimization of deviation thresholds and “digital identity”

In extracting the “digital identities” of CR and SR according to the identification algorithm flow in Section 2.5, we also explored the deviation thresholds for *Rt* and *m/z*. First, we found that the retention times did drift at different acquisition times. However, none of them exceeded 0.10 min. So we set the deviation of *Rt* to Δ*Rt* ≤ 0.10 min. When Δ*m/z* ≤ 0.00 Da, the shared ions from different batches of CR or SR cannot be acquired due to stringent restrictions. When Δ*m/z* ≤ 0.01 Da, the specific ions of CR and SR were all more than 1,000, which allows us to further screen according to ionic strength. When Δ*m/z* ≤ 0.05 Da, there are more specific ions for SR and CR, but in terms of accuracy, Δ*m/z* ≤ 0.05 Da was a great deviation for high-resolution mass spectrometry and showed great identification deviation when matching identification, which would lead to false positive results. Therefore, after comprehensive consideration and reference to the relevant literature, the chosen deviation threshold of *Rt* and *m/z* was Δ*Rt* ≤ 0.10 min and Δ*m/z* ≤ 0.01 Da. In addition, during the specific ion acquisition of SR and CR, we deliberately increased the deviation threshold of *Rt* and *m/z* to Δ*Rt* ≤ 0.20 min and Δm/z ≤ 0.30 Da to remove similar interfering ions to the maximum extent and obtain the corresponding specific ions.

On the other hand, we also investigated the matching situation when the “digital identities” of SR and CR contain 100, 200, and 300 ions. Because of the low price of SR, it is often added to CR to pass itself off as CR. Therefore, we focused on the matching of SR when choosing the number of output ions. When the top 100, 200, and 300 [*Rt*-*m/z*-*I*] were output as “digital identity,” respectively, we examined the matching situation of CR and SR. For example, the MCs of SR (lot number: SR01) with their own “digital identity” were 100.00%, 98.00%, and 99.00%. So we finally chose the top 100 [*Rt*-*m/z*-*I*] to output as “digital identity.” At the same time, the MCs of CR (lot number: CR02) with its own “digital identity” (100 [*Rt*-*m/z*-*I*]) were 86.00%, which meets the analysis requirements.

### 4.3 Discussion of adulteration identification

This study realized the identification of CR and SR and the adulteration analysis based on the “digital identity” of chemical compositions. It also shows the specificity of the “digital identity” of chemical components to some extent. The single Chinese medicine of CR and SR, mixed positive samples, and market CR samples can all be analyzed based on the matching of the “digital identity” of CR and SR chemical components. The optimal number of ion output was determined by the matching situation of the single SR and CR. Based on the MCs of the mixed positive sample feedback, a preliminary detection limit of 54.0% was determined for the MC of SR, and this was used to identify the market MCR07 sample as an adulterated sample. It suggests that the “digital identity” of chemical compositions has some practical value. On the other hand, the accuracy and reliability of the identification based on the “digital identity” of CR and SR’s chemical components were verified by matching mixed positive samples and traditional chemometrics analysis. These are mainly reflected in that based on the matching of the “digital identities” of CR and SR’s chemical components; the SR in the mixed positive samples and the MCR07 samples could be effectively detected, and the four SR-specific chemical components screened by chemometrics analysis could be detected in the SR-containing samples, which was in agreement with the results of the digitized identification analyses. Unfortunately, we were unable to identify exactly what compounds these four differential chemical components are in the chemometric analysis. Regardless, it was used as an assisted verification to verify the identification of CR and SR based on the “digital identity” of chemical compositions, which was feasible and effective.

Moreover, in the chemometric analysis, the 50% SR mixed sample was used as the quality control sample. Using a quality control sample as the reference for peak correction and data transformation, the mass spectrometry data on CR and SR can be integrated into a unified analysis system by converting three-dimensional (3D) LC/MS mass spectra into two-dimensional (2D) data matrices and generating peak intensity lists using Rt and *m/z* data pairs while retaining the data integrity to the greatest extent possible.

On the other hand, since herbs have thousands of chemical components, it is difficult to represent the whole herb by a single or a few chemical components only. So, compared with the traditional chemical marker analysis, “digital identity” can realize the effective use of unknown chemical component groups, no longer limited to a single or a few chemical components, and can improve the exclusivity in the identification and analysis at the individual level of Chinese medicine. At the same time, chemical components tend to have better deliverability in sample processing than in DNA barcoding, which is easily degraded. Digital identities constructed on the basis of chemical constituent groups have better applicability.

### 4.4 Research ideas, strengths, and weaknesses

The general research idea was to control for differences in samples and analysis methods to obtain different results; conversely, the adulteration identification analysis was realized based on the differences in results (Sloop et al., 2023; Jiang et al., 2024; Macias et al., 2023; Ribbenstedt et al., 2018). It had been taken into account that there are also variations in different batches of the same herbs of different origins. However, despite the different origins, batches, and ages, as long as they are the same Chinese medicine, there are bound to be some of the same chemical components. So the shared ions that represent chemical components or specific chemical fragments were screened to maximize the control of sample variation. Furthermore, through unified UHPLC-QTOF-MS^E^ analysis, we controlled the differences in methods. The strengths of this research are that “digital identity” is no longer limited to a single or a few known chemical components and enables the efficient use of unknown components, leading to more accurate and reliable results. It does not need to identify the specific compound structure and molecular formula and can incorporate all the obtained quantized data into the digital matrix to digitally characterize the individual herb, and research focuses on the identification of natural herbs and adulteration at the individual level, rather than chemical compositions in herbs. In addition, the “digital identity” of CR or SR is not changeless. With the increase in samples and the setting of deviation threshold of *Rt* and *m/z*, the “digital identity” can be dynamically adjusted to meet analytical needs. It is worth noting that the number of ions included in the “digital identity” may decrease as the time span and the number of samples in different batches increase or as the bias threshold is adjusted. However, we can still extract the “digital identity” of the same traditional Chinese medicine from different batches at different times and places of origin to obtain the most universal “digital identity.” Moreover, if it can be used for market quality control, we can analyze randomly selected samples from the market at regular intervals and incorporate the data into the “digital identity” collection before using “digital identity” for market identification and analysis. The procedural flow of the algorithm ensures that new data can be added at any time to carry out identification and adulteration analysis. On the other hand, there were also some shortcomings in this study: although all the raw materials used in this study met the requirements of the Chinese Pharmacopoeia, involving different batches and years, the number of samples used in the study was small and in the follow-up study, it is necessary to further increase the sample number to obtain universal results based on the “digital identity” of CR and SR. Moreover, digital identification based on set thresholds is also likely to yield false positive or false negative results, especially in the case of complex market samples. Therefore, additional market samples need to be collected subsequently for further analysis. This also suggests that we can perform an initial digital screening based on the “digital identities” of CR and SR and then analyze samples with MCs around a set threshold for chemometric analysis. In other words, digital preliminary screening combined with precision analysis can be adopted to both increase the efficiency of the analysis and reduce false positive or negative results. In addition, the deviation thresholds still need to be further optimized and can subsequently be accompanied by quality control standards during sample analysis. A reasonable threshold range can be determined based on the actual *Rt* and *m/z* deviation of the QC standards.

## 5 Conclusion

To realize the quick identification and adulteration analysis of CR and SR and improve identification precision, the “digital identities” of CR and SR were constructed based on the shared chemical composition characterization from multiple batches of different origins and the differentiated chemical composition characterization of CR and SR. Based on “digital identity,” the digital identification of single herbs of CR or SR can be realized efficiently and accurately at the individual level with the MC ≥ 86.00%, even if 3% of SR in the mixed samples can still be identified efficiently and accurately. Moreover, 1 of the 12 batches of market samples was identified as an adulterated sample. On the other hand, the accuracy and reliability of CR and SR digital identification based on the “digital identity” of chemical compositions were verified from the perspectives of traditional chemical analysis and chemometrics. It is of great practical significance for improving the efficiency of the identification of SR and CR, cracking down on adulterated drugs, and strengthening the quality control of CR. It can provide a reference for the establishment of the “digital identity” of CR and SR and provide a method to realize the digital identification and adulteration analysis of CR and SR.

## Data Availability

The original contributions presented in the study are included in the article/Supplementary Material; further inquiries can be directed to the corresponding authors.
