# Supplementary material for: Digital identification and adulteration analysis of Codonopsis Radix and Stellariae Radix based on the “digital identity” of chemical compositions
Source: Front Chem. 2024 Nov 7;12:1438321. doi: 10.3389/fchem.2024.1438321 (PMC11579866; doi:10.3389/fchem.2024.1438321)
Supplement: Supplementary file 1 [file Table1.DOCX]

Supplementary Material

# Supplementary Tables

Table S1 The detailed information of herbal materials

| Herbal materials | Batches | Sample Source | Years | Use | Place of origin |
| --- | --- | --- | --- | --- | --- |
| Codonopsis Radix | CR01 | National Institutes for Food and Drug Control | 2012 | "digital identity"; mixed sample | Gansu, China |
| Codonopsis Radix | CR02 | National Institutes for Food and Drug Control | 2015 | Verification; mixed sample | Gansu, China |
| Codonopsis Radix | CR03 | National Institutes for Food and Drug Control | 2019 | "digital identity"; mixed sample | Ningxia, China |
| Codonopsis Radix | CR04 | National Institutes for Food and Drug Control | 2021 | "digital identity"; mixed sample | Shanxi, China |
| Codonopsis Radix | CR05 | National Institutes for Food and Drug Control | 2024 | Verification; mixed sample | Hebei, China |
| Codonopsis Radix | CR06 | National Institutes for Food and Drug Control | 2021 | Verification; mixed sample | Qinghai, China |
| Stellariae Radix | SR01 | Gansu Institutes for Food and Drug Control | 2018 | Verification; mixed sample | Gansu, China |
| Stellariae Radix | SR02 | Gansu Institutes for Food and Drug Control | 2020 | "digital identity"; mixed sample | Ningxia, China |
| Stellariae Radix | SR03 | Gansu Institutes for Food and Drug Control | 2021 | Verification; mixed sample | Shanxi, China |
| Stellariae Radix | SR04 | Gansu Institutes for Food and Drug Control | 2024 | Verification; mixed sample | Ningxia, China |
| Stellariae Radix | SR05 | Gansu Institutes for Food and Drug Control | 2024 | "digital identity"; mixed sample | Liaoning,China |
| Stellariae Radix | SR06 | Gansu Institutes for Food and Drug Control | 2024 | "digital identity"; mixed sample | Gansu, China |
| 0%SR | Mix01 | self-preparation | — | Identification verification | — |
| 3%SR | Mix02 | self-preparation | — | Identification verification | — |
| 5%SR | Mix03 | self-preparation | — | Identification verification | — |
| 10%SR | Mix04 | self-preparation | — | Identification verification | — |
| 20%SR | Mix05 | self-preparation | — | Identification verification | — |
| 30%SR | Mix06 | self-preparation | — | Identification verification | — |
| 40%SR | Mix07 | self-preparation | — | Identification verification | — |
| 50%SR | Mix08 | self-preparation | — | Identification verification | — |
| 100%SR | Mix09 | self-preparation | — | Identification verification | — |
| Market CR samples | MCR01 | Hebei Anguo herbal market | 2024 | Identification analysis | — |
| Market CR samples | MCR02 | Hebei Anguo herbal market | 2024 | Identification analysis | — |
| Market CR samples | MCR03 | Hebei Anguo herbal market | 2024 | Identification analysis | — |
| Market CR samples | MCR04 | Hebei Anguo herbal market | 2024 | Identification analysis | — |
| Market CR samples | MCR05 | Hebei Anguo herbal market | 2024 | Identification analysis | — |
| Market CR samples | MCR06 | Hebei Anguo herbal market | 2024 | Identification analysis | — |
| Market CR samples | MCR07 | Hebei Anguo herbal market | 2024 | Identification analysis | — |
| Market CR samples | MCR08 | Hebei Anguo herbal market | 2024 | Identification analysis | — |
| Market CR samples | MCR09 | Hebei Anguo herbal market | 2024 | Identification analysis | — |
| Market CR samples | MCR10 | Hebei Anguo herbal market | 2024 | Identification analysis | — |
| Market CR samples | MCR11 | Hebei Anguo herbal market | 2024 | Identification analysis | — |
| Market CR samples | MCR12 | Hebei Anguo herbal market | 2024 | Identification analysis | — |

Table S2 The top 100 [*Rt*-*m/z*-*I*] data in "digital identities" of *Codonopsis Radix* (CR)

| Retention time (Rt) | mass-to-charge ratio (m/z) | Ionic strength (I) |
| --- | --- | --- |
| 2.36 | 268.1602 | 94486.1185 |
| 1.29 | 268.1597 | 57658.0434 |
| 5.96 | 350.2037 | 52191.5521 |
| 6.94 | 352.2210 | 35504.7649 |
| 6.35 | 581.2349 | 30744.6532 |
| 2.18 | 254.1447 | 26813.4996 |
| 6.45 | 352.2205 | 24850.2897 |
| 22.15 | 681.4124 | 19802.3704 |
| 7.18 | 419.1781 | 19278.4361 |
| 16.36 | 420.2809 | 18402.7423 |
| 4.93 | 701.2419 | 15826.6078 |
| 25.51 | 992.8230 | 15555.6271 |
| 1.40 | 283.1470 | 14397.6022 |
| 25.15 | 615.4753 | 13911.4754 |
| 23.37 | 657.4132 | 12816.3593 |
| 17.08 | 991.6956 | 12023.0469 |
| 22.90 | 483.3931 | 11741.8925 |
| 17.81 | 404.2863 | 11428.4866 |
| 25.66 | 1016.8404 | 10766.4327 |
| 4.82 | 366.1970 | 9956.4056 |
| 23.35 | 689.5092 | 9801.2141 |
| 3.48 | 382.1565 | 9596.3057 |
| 22.88 | 645.4824 | 9354.6426 |
| 4.41 | 310.1344 | 9190.3192 |
| 1.11 | 230.1439 | 9022.5569 |
| 6.35 | 597.2088 | 9018.0389 |
| 12.79 | 231.1427 | 8547.2771 |
| 20.67 | 843.4716 | 8498.1286 |
| 20.99 | 681.4138 | 8431.8919 |
| 1.13 | 270.1402 | 8372.5040 |
| 25.25 | 1034.8409 | 8369.2007 |
| 5.30 | 368.2148 | 7334.9666 |
| 1.07 | 326.1304 | 7321.3559 |
| 19.26 | 293.2535 | 7104.2650 |
| 7.18 | 199.1163 | 7099.0678 |
| 25.56 | 1153.3093 | 6938.1870 |
| 22.89 | 889.7187 | 6881.7176 |
| 1.33 | 427.1906 | 6849.0565 |
| 5.69 | 743.2938 | 6744.4093 |
| 17.24 | 468.3198 | 6727.7904 |
| 16.33 | 492.3202 | 6637.1340 |
| 6.47 | 350.2025 | 6625.3452 |
| 22.12 | 657.4142 | 6541.9577 |
| 1.90 | 416.2009 | 6231.8786 |
| 6.35 | 199.1163 | 6184.8687 |
| 14.88 | 233.1582 | 6134.1853 |
| 23.88 | 364.3669 | 5743.1996 |
| 7.19 | 815.3647 | 5638.1319 |
| 19.80 | 277.2219 | 5537.3662 |
| 7.18 | 435.1515 | 5475.3243 |
| 23.80 | 761.4927 | 5401.1585 |
| 5.53 | 449.2097 | 5399.4964 |
| 1.06 | 392.1683 | 5144.1194 |
| 5.70 | 338.2039 | 5004.4881 |
| 16.38 | 231.1425 | 4918.9558 |
| 5.82 | 368.2150 | 4888.7713 |
| 6.05 | 419.1982 | 4866.3863 |
| 25.08 | 671.5066 | 4768.1017 |
| 15.70 | 349.2420 | 4723.9452 |
| 20.01 | 277.2205 | 4678.8838 |
| 23.48 | 557.4358 | 4612.2748 |
| 1.07 | 408.1613 | 4431.8014 |
| 4.78 | 611.2663 | 4406.0261 |
| 4.93 | 717.2156 | 4221.3076 |
| 15.95 | 442.3233 | 3904.6672 |
| 25.38 | 1000.8314 | 3872.4983 |
| 6.58 | 581.2344 | 3742.7377 |
| 15.98 | 616.3605 | 3738.6953 |
| 21.27 | 279.2377 | 3602.9457 |
| 24.67 | 891.7309 | 3533.1239 |
| 2.90 | 297.1607 | 3417.6838 |
| 1.13 | 456.2124 | 3266.6079 |
| 7.58 | 487.3606 | 3219.4442 |
| 6.19 | 265.1014 | 3201.3056 |
| 20.97 | 552.4667 | 3113.8865 |
| 14.85 | 699.3730 | 3062.6480 |
| 16.35 | 619.3006 | 3058.0673 |
| 12.53 | 640.4041 | 3029.7632 |
| 25.98 | 613.4713 | 3021.2123 |
| 7.46 | 331.1590 | 2912.1961 |
| 17.34 | 595.3022 | 2880.3749 |
| 21.85 | 975.5932 | 2872.9242 |
| 21.37 | 483.3919 | 2848.9367 |
| 17.39 | 482.3361 | 2788.6867 |
| 20.24 | 277.2218 | 2767.9873 |
| 24.95 | 340.3667 | 2682.1367 |
| 19.61 | 424.3149 | 2634.8119 |
| 21.55 | 633.4854 | 2592.5201 |
| 25.87 | 591.4736 | 2591.2658 |
| 2.84 | 382.1588 | 2550.6510 |
| 7.18 | 416.1695 | 2543.1639 |
| 1.06 | 240.1255 | 2314.9161 |
| 23.65 | 684.5377 | 2281.7444 |
| 1.20 | 248.1537 | 2230.1593 |
| 6.35 | 155.0888 | 2228.1942 |
| 8.46 | 416.3450 | 2189.5819 |
| 3.94 | 303.1397 | 2186.4101 |
| 19.80 | 315.2559 | 2168.2024 |
| 1.91 | 430.2142 | 2129.5407 |
| 25.98 | 1008.8201 | 2108.1468 |

Table S3 The top 100 [Rt-m/z-I] data in "digital identities" of and Stellariae Radix (SR)

| Retention time (*Rt*) | mass-to-charge ratio (*m/z*) | Ionic strength (*I*) |
| --- | --- | --- |
| 9.55 | 720.4427 | 215334.7370 |
| 11.73 | 903.5257 | 197876.2880 |
| 4.63 | 565.1738 | 112183.3954 |
| 9.69 | 983.5212 | 110458.4312 |
| 8.94 | 384.1329 | 100744.1642 |
| 14.41 | 411.1696 | 95791.1305 |
| 11.03 | 1032.5998 | 75433.1548 |
| 11.99 | 1012.5821 | 67128.2070 |
| 20.47 | 676.4360 | 63022.4125 |
| 19.55 | 662.4199 | 52811.3233 |
| 16.97 | 480.1924 | 51705.0229 |
| 9.27 | 637.3548 | 49899.9001 |
| 24.45 | 1104.8699 | 47392.3600 |
| 5.93 | 741.2262 | 45341.2557 |
| 4.93 | 565.1733 | 44398.0722 |
| 25.18 | 972.8210 | 39022.3597 |
| 13.02 | 340.1408 | 37539.4952 |
| 23.14 | 901.7570 | 37323.3998 |
| 5.18 | 595.3195 | 36156.7010 |
| 24.20 | 1090.8526 | 29905.4599 |
| 4.38 | 227.0894 | 27552.5251 |
| 17.60 | 424.1976 | 26808.1639 |
| 6.95 | 578.8304 | 25813.6594 |
| 5.77 | 937.3055 | 25703.7118 |
| 19.24 | 520.2248 | 25564.5919 |
| 11.97 | 269.1009 | 24413.8420 |
| 17.81 | 439.2261 | 23912.6164 |
| 12.24 | 931.5580 | 23877.2300 |
| 10.76 | 502.1985 | 22713.7247 |
| 11.28 | 340.1398 | 22071.2118 |
| 17.59 | 322.1293 | 19879.6592 |
| 7.09 | 303.1078 | 19749.7478 |
| 11.74 | 941.4824 | 17862.1617 |
| 9.56 | 346.7282 | 17358.3133 |
| 25.25 | 866.7043 | 16965.1366 |
| 7.19 | 919.2947 | 16955.4194 |
| 6.05 | 373.1401 | 16471.7812 |
| 16.85 | 410.1840 | 15654.0115 |
| 23.26 | 988.8128 | 15348.1774 |
| 18.31 | 629.3703 | 15297.1560 |
| 18.68 | 530.2067 | 14662.9849 |
| 10.14 | 338.1240 | 14647.0068 |
| 9.69 | 964.5810 | 14014.4865 |
| 15.92 | 422.1845 | 13382.2341 |
| 19.78 | 943.7302 | 12862.6187 |
| 13.39 | 338.1244 | 12805.8069 |
| 10.75 | 271.0799 | 12770.9035 |
| 10.26 | 285.0964 | 12524.2787 |
| 15.35 | 438.1796 | 12197.2366 |
| 12.07 | 873.5145 | 12029.8321 |
| 20.59 | 443.3636 | 12025.9684 |
| 18.20 | 893.3039 | 11944.7620 |
| 4.38 | 181.0821 | 11565.4001 |
| 8.99 | 254.1002 | 11426.2038 |
| 22.56 | 1120.8616 | 11277.3823 |
| 5.59 | 562.1843 | 10994.0145 |
| 8.44 | 517.1979 | 10302.8590 |
| 6.05 | 575.1913 | 9925.4456 |
| 25.55 | 645.4989 | 9860.9215 |
| 9.55 | 607.3550 | 9718.3858 |
| 21.58 | 707.5258 | 9431.7283 |
| 9.69 | 1021.4779 | 9211.4383 |
| 22.96 | 1134.8799 | 9041.0637 |
| 1.35 | 203.0880 | 8803.0199 |
| 7.46 | 400.1264 | 8737.7601 |
| 4.36 | 595.1851 | 8729.0351 |
| 4.37 | 595.1839 | 8712.3588 |
| 18.32 | 427.3192 | 8678.0526 |
| 13.29 | 426.1429 | 8633.0783 |
| 14.18 | 279.2407 | 8449.1318 |
| 15.68 | 480.1917 | 8318.2699 |
| 12.45 | 354.1192 | 8304.0443 |
| 5.24 | 535.1617 | 8181.7565 |
| 9.34 | 470.3113 | 7976.0087 |
| 14.41 | 843.3128 | 7967.0204 |
| 11.28 | 701.2550 | 7827.6009 |
| 22.91 | 705.5217 | 7496.6262 |
| 23.36 | 549.3955 | 7458.1735 |
| 10.15 | 733.2445 | 7438.3937 |
| 15.90 | 438.1794 | 7245.8061 |
| 8.94 | 789.2385 | 7195.1060 |
| 12.96 | 371.1714 | 7006.9951 |
| 14.54 | 413.1831 | 6959.1783 |
| 17.59 | 460.1413 | 6955.0618 |
| 22.27 | 1106.8466 | 6953.1738 |
| 8.99 | 227.0882 | 6488.6723 |
| 9.28 | 675.3113 | 6421.3523 |
| 17.59 | 290.1016 | 6418.1300 |
| 6.37 | 579.3227 | 6244.8708 |
| 9.36 | 518.1928 | 6198.6271 |
| 23.21 | 897.6022 | 5959.3132 |
| 9.68 | 987.5617 | 5958.5789 |
| 23.14 | 575.5224 | 5616.0212 |
| 3.95 | 727.2323 | 5562.3529 |
| 12.19 | 338.1607 | 5499.7076 |
| 7.15 | 449.1695 | 5468.8526 |
| 4.48 | 889.2910 | 5373.3532 |
| 23.51 | 605.4350 | 5273.1618 |
| 4.56 | 727.2326 | 5252.4165 |
| 8.09 | 607.3535 | 5218.4072 |
